# Maternal effects and urbanization: Variation of yolk androgens and immunoglobulin in city and forest blackbirds

**DOI:** 10.1002/ece3.6058

**Published:** 2020-02-04

**Authors:** Jesko Partecke, Gergely Hegyi, Patrick S. Fitze, Julien Gasparini, Hubert Schwabl

**Affiliations:** ^1^ Department of Migration Max Planck Institute of Animal Behavior Radolfzell Germany; ^2^ Department of Biology University of Konstanz Konstanz Germany; ^3^ School of Biological Sciences and Center for Reproductive Biology Washington State University Pullman Pullman WA USA; ^4^ Department of Systematic Zoology and Ecology Eötvös Loránd University Budapest Hungary; ^5^ Museo Nacional de Ciencias Naturales (MNCN‐CSIC) Madrid Spain; ^6^ Sorbonne Université UPEC CNRS INRA IRD Institut d'Ecologie et des Sciences de l'Environnement de Paris Paris France

**Keywords:** anthropogenic environment, epigenetic developmental modification, local adaptation, maternal antibodies, maternal hormones, micro‐evolution, natural selection, phenotypic plasticity

## Abstract

Wildlife inhabiting urban environments exhibit drastic changes in morphology, physiology, and behavior. It has often been argued that these phenotypic responses could be the result of micro‐evolutionary changes following the urbanization process. However, other mechanisms such as phenotypic plasticity, maternal effects, and developmental plasticity could be involved as well. To address maternal effects as potential mechanisms, we compared maternal hormone and antibody concentrations in eggs between city and forest populations of European blackbirds (*Turdus merula*), a widely distributed species for which previous research demonstrated differences in behavioral and physiological traits. We measured egg and yolk mass, yolk concentrations of androgens (androstenedione [A_4_], testosterone [T], 5α‐dihydrotestosterone [5α‐DHT], and immunoglobulins [IgY]) and related them to population, clutch size, laying order, embryo sex, and progress of breeding season. We show (a) earlier onset of laying in the city than forest population, but similar egg and clutch size; (b) higher overall yolk androgen concentrations in the forest than the city population (sex‐dependent for T); (c) greater among‐female variation of yolk T and 5α‐DHT concentrations in the forest than city population, but similar within‐clutch variation; (d) similar IgY concentrations with a seasonal decline in both populations; and (e) population‐specific positive (city) or negative (forest) association of yolk A_4_ and T with IgY concentrations. Our results are consistent with the hypotheses that hormone‐mediated maternal effects contribute to differences in behavioral and physiological traits between city and forest individuals and that yolk androgen and immunoglobulin levels can exhibit population‐specific relationships rather than trade‐off against each other.

## Graphical abstract

Urbanization of animals is often associated with phenotypic alterations. These alterations are mainly discussed in the framework of adaptation or phenotypic plasticity. Maternal effects have been neglected so far. Our study highlights for the first time the potential of maternal effects in the urbanization process of animals.

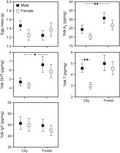

## 1 INTRODUCTION

Animals inhabiting urban environments are characterized by marked differences in morphology, physiology, and behavior from conspecifics living in less urbanized habitats (Alberti et al., 2017). These alterations could be the result of genetic drift, local adaptation, immigrant selection, and phenotypic plasticity (Johnson & Munshi‐South, 2017; Partecke, 2014). The special form of developmental plasticity associated with maternal effects has not been addressed much in the identification and discussion of the processes that lead to expression of different phenotypes in urban versus rural individuals. While common garden studies have shown that differences in some behavioral, physiological, and life history traits between city and forest individuals are intrinsic, excluding phenotypic plasticity as the primary explanatory mechanism (Atwell et al., 2012; Costantini, Greives, Hau, & Partecke, 2014; Miranda, Schielzeth, Sonntag, & Partecke, 2013; Partecke & Gwinner, 2007; Partecke, Schwabl, & Gwinner, 2006), these studies cannot rule out maternal effects and organizational effects during development.

Hormone‐mediated maternal effects are powerful mechanisms of developmental modification of offspring phenotype across vertebrates (Dantzer et al., 2013; Giesing, Suski, Warner, & Bell, 2010; Groothuis & Schwabl, 2008; Meylan, Miles, & Clobert, 2012). For example, androgens deposited by female birds into their eggs affect multiple and diverse aspects of offspring development and phenotype, with effects becoming evident during early life and some lasting into adulthood (Groothuis & Schwabl, 2008; Schwabl & Groothuis, 2010). Environmental conditions known to influence the concentrations of androgens in the avian egg include breeding density (e.g., Duckworth, Belloni, & Anderson, 2015), nest predation risk (e.g., Schwabl, Mock, & Gieg, 1997), parasite prevalence (e.g., Tschirren, Richner, & Schwabl, 2004), and food abundance (e.g., Morosinotto et al., 2016; Verboven, Monaghan, Evans, & Schwabl, 2003), all of which factors often also differ starkly between urban and more natural habitats (Shochat, Warren, Faeth, McIntyre, & Hope, 2006).

Yolk androgen manipulations (mainly T and/or A4) in various species revealed permanent effects on the phenotype of adult offspring, including such traits as neophobia (Tobler & Sandell, 2007), agonistic and sexual behavior (Eising, Muller, & Groothuis, 2006; Partecke & Schwabl, 2008), and stress response (H. Schwabl & J. Partecke, in preparation), traits that often also differ between urban and rural individuals (Atwell et al., 2012; Gil & Brumm, 2014; Møller, 2010), including the city and forest populations of the European blackbird studied here. Previous common garden experiments with individuals originating from the populations of blackbirds that we studied here for maternal effects have shown differences in timing of reproduction (Partecke, Van't Hof, & Gwinner, 2004), tendency to migrate (Partecke & Gwinner, 2007), adrenocortical stress response (Partecke et al., 2006), and behavioral style related to personality (Miranda et al., 2013). While these studies point to intrinsic genetic differences, their design does, however, not allow to exclude early developmental effects, in particular hormone‐mediated maternal effects. To address whether androgen‐mediated maternal effects contribute to the development of phenotype differences between individuals of city and forest populations of European blackbirds, we investigated, as a first step, if exposure of the embryo to maternal androgens in the eggs differs between the populations.

We included measurement of immunoglobulin concentrations in the yolks of these eggs to address if and how different mediators of maternal effects trade off or are integrated with each other. The egg is a repository of maternal antibodies (Grindstaff, Brodie, & Ketterson, 2003), such as the avian immunoglobulin IgY, which can reduce chick susceptibility to parasites and provide passive protection to naive embryos (Grindstaff et al., 2003). The transmission of immunity mediators from mother into egg could simply reflect pathogen exposure levels of the egg‐forming female or be integrated with other maternal effect mediators such as hormones to result in optimally prepared offspring phenotype for certain environments and conditions (Giraudeau & Ducatez, 2016; Postma, Siitari, Schwabl, Richner, & Tschirren, 2014). Integration may, however, be constrained by physiological limitations, trade‐off, and costs (Muehlenbein, Prall, & Peck, 2017). The transmission of androgens and immune compounds may antagonistically trade‐off against each other in the mother (Okuliarova et al., 2014), if immune challenge and antibody production reduces steroid hormone production. Or, vice versa, androgens may inhibit immune system activation and antibody production (Foo, Nakagawa, Rhodes, & Simmons, 2017). The latter may also apply to the offspring. Infestation with certain ectoparasites has been shown to be associated with reduced yolk androgen levels (Tschirren et al., 2004), consistent with some antagonistic trade‐off between immune activation and androgen production or transmission. Consistent with the pattern found in pair‐wise comparisons of 11 urban and rural populations of European blackbirds (Evans, Gaston, Sharp, et al., 2009), blood parasite prevalence in two previous study years was higher in our forest population than in our city population, and it increased with progress of the season in the forest population (Geue & Partecke, 2008). Based on these lines of evidence, we predicted higher and seasonally increasing IgY titers in eggs of forest compared to city birds, coupled with lower yolk androgens in the forest than the city eggs if antagonistic trade‐off were at work.

## 2 MATERIALS AND METHODS

All procedures were performed in accordance with the German regulation on animal experimentation (approval by Ethical Committee of Bavaria; reference number: 211‐2531‐30/99).

### 2.1 Field methods and sampling

We studied European blackbirds in a cemetery (Alter Südfriedhof) in the city center of Munich, Germany (48°07′N, 11°34′E; 518 m asl) and in a nearby (approx. 40 km as the crow flies) rural woodland (Raisting: 47°53′N, 11°04′E; 553 m asl). These study areas differ markedly in exposure levels to humans, human settlement, traffic, nesting sites, and artificial light exposure (Dominoni, Carmona‐Wagner, Hofmann, Kranstauber, & Partecke, 2014; Partecke et al., 2006) and are representative for the abiotic and biotic conditions typical for parkland, gardens, and cemeteries of central European inner‐city habitat versus surrounding rural land. Nesting sites in the cemetery were mainly in ivy (*Hedera* spp.) covering tombstones and cemetery walls as well as small shrubs. Nesting sites in rural woodland which is characterized by temperate‐mixed deciduous forest, with alder (*Alnus* spp.) and spruce (*Picea* spp.) as dominant species, were small trees, bushes, and shrubs. In both sites, hereafter referred to as “city” and “forest,” we searched for freshly built nests during the entire breeding period (March–June) in 2002 and checked nests once a day for egg laying. Freshly laid eggs were marked with nontoxic ink and immediately replaced with a dummy egg (http://www.graf-versand.de) to ensure normal female laying behavior. We collected entire clutches in this way.

We identified first clutches laid at the beginning of the breeding season at each site, but we could not distinguish between replacement and second or third clutches among later laid clutches. Therefore, we divided the clutches into two batches: first and later clutches. City first clutches (*N* = 12) were collected between March 12 and 27 and forest first clutches (*N* = 15) between March 21 and April 13. City later clutches (*N* = 9) were collected between April 24 and May 18 and forest later clutches (*N* = 8) between April 24 and May 20. In total, we obtained data on yolk steroid (A_4_, T, 5α‐DHT) concentrations from 168 eggs, embryonic sex for 44 cities and 47 forest eggs of first clutches, and 41 city and 36 forest eggs of later clutches. For the IgY levels, we analyzed 60 eggs from 15 city nests and 56 eggs from 16 forest nests. Because females were not individually banded, we could not distinguish between adult and young females and between subsequent clutches laid by the same females. To minimize the chance that subsequent clutches originated from the same females, we searched for new nests in different areas of each site later in the season. Immediately after collection, eggs were weighed (to the nearest 0.01 g), measured (length and maximum width, to the nearest 0.05 mm), and subsequently placed in an incubator at 37.5°C with 60% humidity to allow embryonic development for 96 hr. Thereafter, embryo and yolk were separated. In order to facilitate an easy separation of the embryo and yolk from the albumen, we placed the whole egg for 1 hr at −80°C. Yolk mass was weighed, and embryo and yolk were stored at −80°C until further analysis.

### 2.2 Hormone analyses

Androstenedione (A_4_), 5α‐dihydrotestosterone (5α‐DHT), and testosterone (T) concentrations in yolk were quantified using described separation protocols (Schwabl, 1993) and radioimmunoassays (Wingfield & Farner, 1975). Approximately 200 mg of homogenized yolk was diluted with 200 µl distilled water. After adding 20 µl (2,000 cpm) tritiated A_4_, 5α‐DHT, T, and 17β‐estradiol (E_2_) for calculation of recoveries, samples were extracted twice with 4 ml petroleum ether/diethyl ether (30/70%), followed by precipitation of lipids with 90% ethanol. Then, hormones were separated on diatomaceous earth chromatography columns. Briefly, samples were reconstituted in 10% ethyl acetate in 2,2,4‐trimethylpentane and transferred to the columns. A_4_ was eluted with 2% ethyl acetate in 2,2,4‐trimethylpentane, 5α‐DHT with 10% ethyl acetate, T with 20% ethyl acetate, and E_2_ with 40%. Hormone concentrations were measured in double competitive‐binding radioimmunoassays (RIA) with ^3^H‐labeled steroids: NET 553 (T), NET 544 (5α‐DHT), NET 469 (A4), NET‐517 (E_2_), obtained from PerkinElmer Life and Analytical Sciences. Antibodies used were as follows: T 3003 (Wien Laboratories) for both T and 5α‐DHT, A 1707 (Wien Laboratories) for A_4_, and AR1702 (Biogenesis) for E_2_. Average recoveries were 65.1% for A_4_, 41.1% for DHT, 68.4% for T, and 61% for E_2_. Recoveries for 5α‐DHT are notoriously low across species. However, 5α‐DHT levels were well above assay detection limit (2 pg/tube) in all samples and therefore the modest recoveries did not inflate calculated concentrations. Mean intra‐assay variation was 8.5% for A_4_, 12.3% for DHT, and 8.4% for T. Inter‐assay variation ranged between 2.5% and 6.6%. Detection limits were 0.08 pg/mg yolk for A_4_, 0.06 pg/mg for DHT, and 0.04 pg/mg for T. We collected the chromatography fractions for E_2_, but E_2_ levels were below assay detection limit (1.95 pg/tube, i.e., 0.05 pg/mg yolk for an average recovery of 61%) in most samples and are therefore not further considered. Since incubation can alter yolk steroid concentrations (Elf & Fivizzani, 2002; Gilbert, Bulmer, Arnold, & Graves, 2007; Paitz, Bowden, & Casto, 2011), the reported androgen concentrations do not reflect initial female allocation, but rather relative differences in exposure levels of embryos to steroids. Moreover, recent studies have reported significant changes in yolk hormone concentrations between ovulation and oviposition (Kumar et al., 2018). Hence, hormone amounts in even freshly laid eggs may not adequately reflect maternal allocation.

### 2.3 Immunoglobulin assay

Yolk IgY concentration was determined using an enzyme‐linked immunosorbent assay (ELISA) sandwich technique, following the protocol of (Gasparini et al., 2007). Briefly, 0.5 g of egg yolk was diluted in 0.5 g of PBS (1:1 dilution). About 100 μl chloroform was added to 100 μl of this dilution, vigorously mixed for 1 min, and thereafter centrifuged for 6 min. The clear supernatant was used for IgY concentration determination. Then, we followed the sandwich ELISA described in Gasparini et al. (2007) for total IgY concentration. Optical density (OD) was used for statistical analyses (Fitze, Tschirren, Gasparini, & Richner, 2007), because all samples had been treated identically. We used 20 and 24 repeated samples, respectively, to calculate inter‐ and intraplate repeatability (interplate *r* = .90; intraplate: *r* = .97).

### 2.4 Sex determination

Embryo sex was identified by amplification of the intron of the CHD1 genes on the sex chromosomes, following standard procedures (Griffiths, Double, Orr, & Dawson, 1998).

### 2.5 Statistical analyses

Yolk steroid and IgY concentrations were log transformed before analysis to obtain normality. Statistical models without random factors were fit in Statistica 5.5 (StatSoft, Inc.), while general linear mixed models were calculated in the lmerTest procedure of R (Kuznetsova, Brockhoff, & Christensen, 2017), using Satterthwaite approximation of degrees of freedom. We conducted backward stepwise model selection with reintroduction throughout (Hegyi & Laczi, 2015). To test for differences between the city and forest population in laying date and clutch size, we performed general linear models using population and clutch batch (first and later clutches) as fixed effects together with their interaction. Binomial models with number of males as the dependent variable and clutch size as a binomial denominator were used to test for differences in clutch sex ratios (% males) between city and forest clutches. The binomial model with the population × clutch batch interaction did not converge. Therefore, we performed the models separately for first and later clutches. To test for differences in egg parameters between the two populations, we used general linear mixed models with egg mass, hormone, or IgY levels as dependent variable (separate models for each) and nest identity as a random effect. Fixed factors were clutch batch (first vs. later clutches), population (city vs. forest), and sex, while continuous predictors were laying order and laying date. As clutch size generally ranged from 3 to 6 eggs (with a single two‐egg clutch), laying order was coded as first, middle, and last egg (a clutch of four eggs, for example, would have a laying order of one first, two middle, and one last egg). Laying date was used as a residual from the above general linear model to account for a batch‐specific population effect. We also included the two‐way interactions of population, sex, and laying order. Yolk mass correlated with egg mass (*N* = 166, Pearson's *r* = .32 *p* < .001) and therefore only egg mass was included in analyses.

Due to assumed population differences in parasite load (see Section 1), we chose to analyze the causal chain of relationship from immunoglobulins (presumably affected by parasites) to androgens/macronutrients and accordingly constructed the general linear models used to examine population‐specific relationships of egg mass or maternal hormones (yolk A_4_, T, 5α‐DHT levels) with IgY levels across city and forest clutches by using egg mass/hormone as dependent variable and IgY as independent variable. To account for nest effects, we used average values for each clutch. Fixed factors were population and clutch batch (first and later clutches). To avoid collinearity with factors, we extracted residual IgY levels from the above described general linear models to account for seasonal change. We also added the interactions of IgY with population and batch. All tests were two‐tailed, significance level was set at *α* = .05, and data are presented as mean ± *SE*, if not otherwise mentioned. Transformed data are graphically represented as back‐transformed means ± *SE*, resulting in asymmetric representation of the error bars.

## 3 RESULTS

### 3.1 Laying date, clutch size, and sex ratio

City blackbirds laid their first clutches earlier than forest blackbirds, while later clutches were laid (i.e., collected) approximately at the same time. First clutches were smaller than later clutches. Clutches tended to be larger in the city than in the forest. Sex ratio did not differ among populations, neither in first nor in later clutches (Table 1).

**Table 1:** Mean laying date (Julian date ± *SE*), clutch size (±*SE*) and sex ratio (% males) in the city and the forest European blackbird population and test statistics

	First clutch		Later clutch		Terms	*F*	*df*
	City *N* = 12	Forest *N* = 15	City *N* = 9	Forest *N* = 8			
Laying date	79 ± 1	94 ± 1	125 ± 2	132 ± 3	Clutch batch	**508.1** ***	**1, 40**
					Population	**39.7** ***	**1, 40**
					Population × clutch batch	**5.3** *	**1, 40**
Clutch size	3.7 ± 0.1	3.1 ± 0.1	4.5 ± 0.3	4.5 ± 0.2	Clutch batch	**35.6** ***	**1, 42**
					Population	3.5	1, 40
					Population × clutch batch	0.6	1, 40
						Wald chi^2^	*df*
Sex ratio	0.36	0.59	0.53	0.48	Population (First clutch)	0.4	1
					Population (Later clutch)	2.1	1

Note

Significant effects are in boldface.

* *p* < .05,

*** *p < *.001.

### 3.2 Egg size

Egg mass was not related to population, sex, or laying date, but it increased with laying order in both populations (Table 2, Figure 2). Egg mass was unrelated to residual IgY concentration or its interactions with population or clutch batch (first vs. later) (Table 2).

**Table 2:** Variation in egg mass and egg components (Androstenedione [A_4_], dihydrotestosterone [DHT], testosterone [T], and immunoglobulin Y concentration [IgY]) of European blackbird clutches in relation to early versus late clutches (batch), city versus forest population (pop), embryo sex (sex), laying order (lay ord), and residual laying date within batch (res lay date)

	Egg mass		A_4_		DHT		T		IgY	
	*F*	*df*	*F*	*df*	*F*	*df*	*F*	*df*	*F*	*df*
Batch	4.04	1, 41.78	<0.01	1, 40.06	0.67	1, 36.50	<0.01	1, 38.23	**15.57** ***	**1, 27.14**
Pop	0.04	1, 41.93	**3.98** *	**1, 40.82**	**4.99** *	**1, 38.07**	2.14	1, 39.38	<0.01	1, 25.16
Sex	0.13	1, 121.57	0.87	1, 128.59	0.28	1, 128.60	1.81	1, 123.51	0.08	1, 96.88
Lay ord	**15.73** **	**1, 121.22**	**9.09** **	**1, 120.83**	1.69	1, 117.38	**5.36** *	**1, 116.61**	0.01	1, 82.67
Res lay date	0.10	1, 42.12	1.04	1, 40.87	**6.58** *	**1, 38.48**	1.29	1, 38.48	0.11	1, 28.21
Pop × sex	1.86	1, 120.33	0.17	1, 128.30	0.07	1, 127.66	**4.58** *	**1, 123.45**	0.14	1, 95.53
Pop × lay ord	<0.01	1, 120.21	0.13	1, 120.08	0.55	1, 116.36	0.24	1, 115.60	0.12	1, 81.31
Sex × lay ord	1.82	1, 123.06	0.17	1, 129.93	1.06	1, 128.79	0.96	1, 123.84	1.45	1, 108.11

Note

General linear mixed models with backward stepwise parameter selection with reintroduction and Satterthwaite approximation for error *df* Clutch was included as a random factor (always significant, not shown here). Significant effects are in boldface.

* *p* < .05,

** *p* < .01,

*** *p* < .001.

### 3.3 Yolk androgens

**Figure 1:**
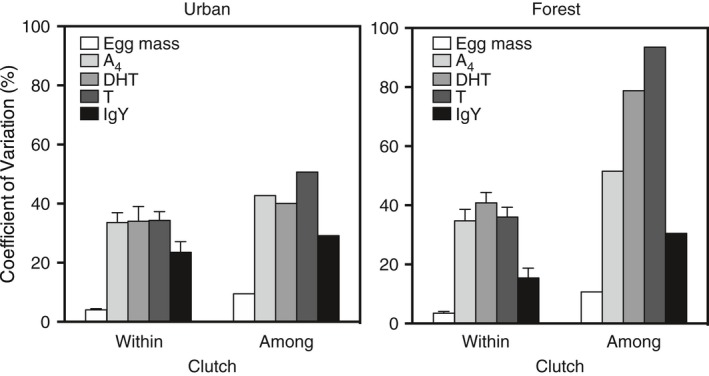
Within‐ and among‐clutch variation (coefficient of variation: CV = 100 × standard deviation/mean) in egg mass, yolk androgen concentration, and immunoglobulin (IgY) concentration in a city and forest population of the European blackbird

The androgens of the synthesis pathway from A_4_ to T to 5α‐DHT were positively correlated with their respective precursor for individual eggs and clutch means in the forest population, but only for individual eggs and not clutch means in the city population: Pearsons *r*: individual eggs: T with its precursor A_4_: city *r* = .6364, *p* < .001; forest *r* = .740, *p* < .001; 5α‐DHT with its precursor T: city *r* = .257, *p* < .05; forest *r* = .373, *p* < .001; clutch means: T with its precursor A_4_: city *r* = .323, *p* = .115; forest *r* = .802, *p* < .001; 5α‐DHT with its precursor T: city *r* = .015, *p* = .945; forest *r* = .468, *p* < .05). Within‐clutch variation, defined as the coefficient of variation (CV), in yolk A_4_, T, and 5α‐DHT concentrations, did not differ between the city and the forest population, while among‐clutch variation (CV) was larger in the forest than the city (Figure 1).

**Figure 2:**
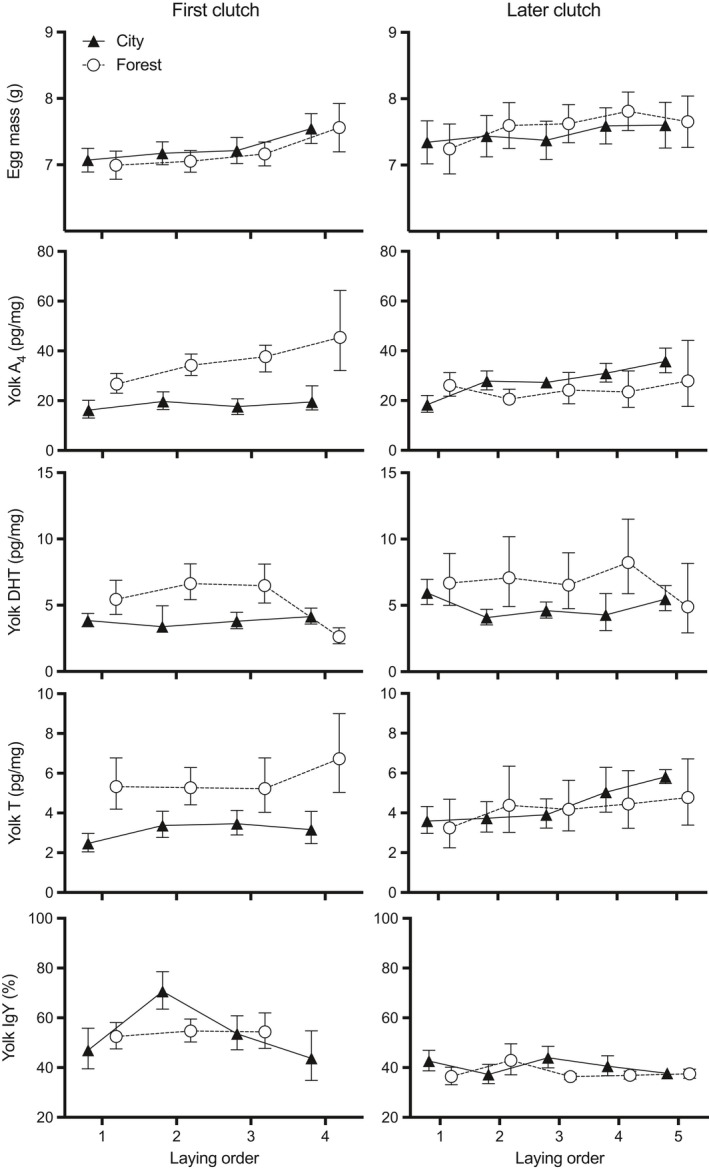
Variation of egg mass, yolk A_4_, DHT, T, and IgY concentrations (mean ± *SE*) in relation to laying order in first (left) and later (right) clutches in a city and a forest population of the European blackbird. Data of yolk A_4_, DHT, T, and IgY levels are back‐transformed means resulting in asymmetrical SE. A4, androstenedione; DHT, dihydrotestosterone; IgY, immunoglobulins; T, testosterone

**Figure 3:**
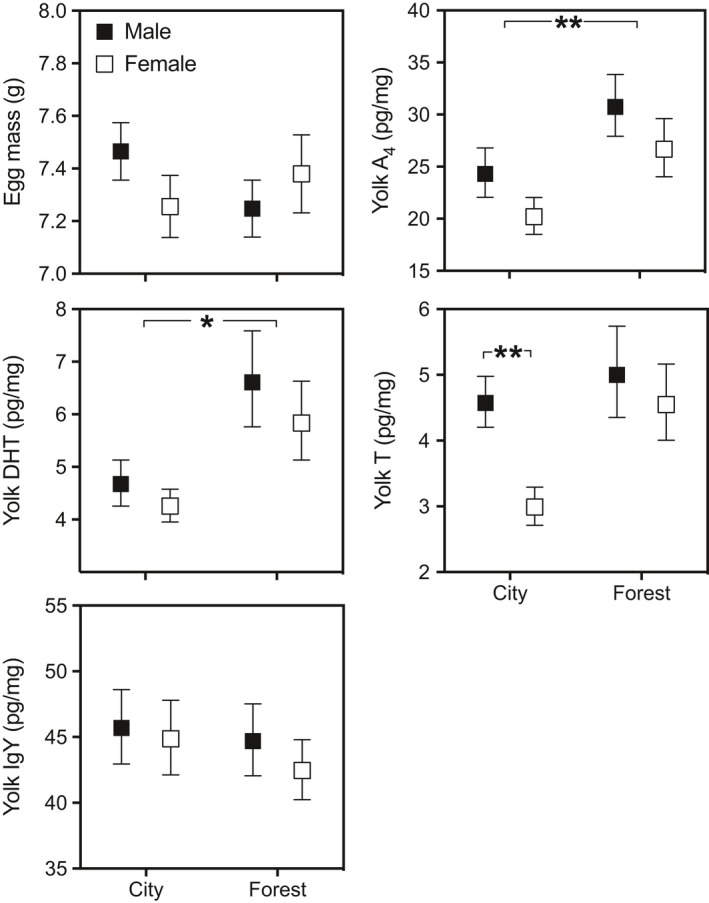
Variation of egg mass and yolk A_4_, DHT, T, and IgY concentrations (mean ± *SE*) in relation to the embryonic sex in a city and a forest population of the European blackbird. Data of yolk A_4_, DHT, T, and IgY levels are back‐transformed means resulting in asymmetrical *SE*. *p < .05, **p < .01. A_4_, androstenedione; DHT, dihydrotestosterone; IgY, immunoglobulins; T, testosterone

Yolk A_4_ marginally and 5α‐DHT significantly differed between populations, with higher levels in forest eggs (Table 2; Figure 2). T concentrations showed an interaction between population and sex: in city clutches, female eggs had significantly lower yolk T levels than male eggs; in contrast, they did not differ between the sexes in the forest (Figure 3; Table 2). Yolk A_4_ and T significantly increased with laying order (Figure 2; Table 2), but 5α‐DHT did not. Embryonic sex had no effect on yolk steroids other than its interaction with population for T (see above). 5α‐DHT was negatively related to residual (within batch) laying date.

### 3.4 Immunoglobulins

Immunoglobulins concentrations did not differ between the forest and city population and were unrelated to any other parameter except for clutch batch (Table 2). In both populations, there was a seasonal decline in IgY concentrations, with eggs of later clutches showing lower concentrations than eggs of first clutches (*F*_1,31_ = 11.5, *p* < .01).

### 3.5 Yolk androgen and IgY relationship

**Figure 4:**
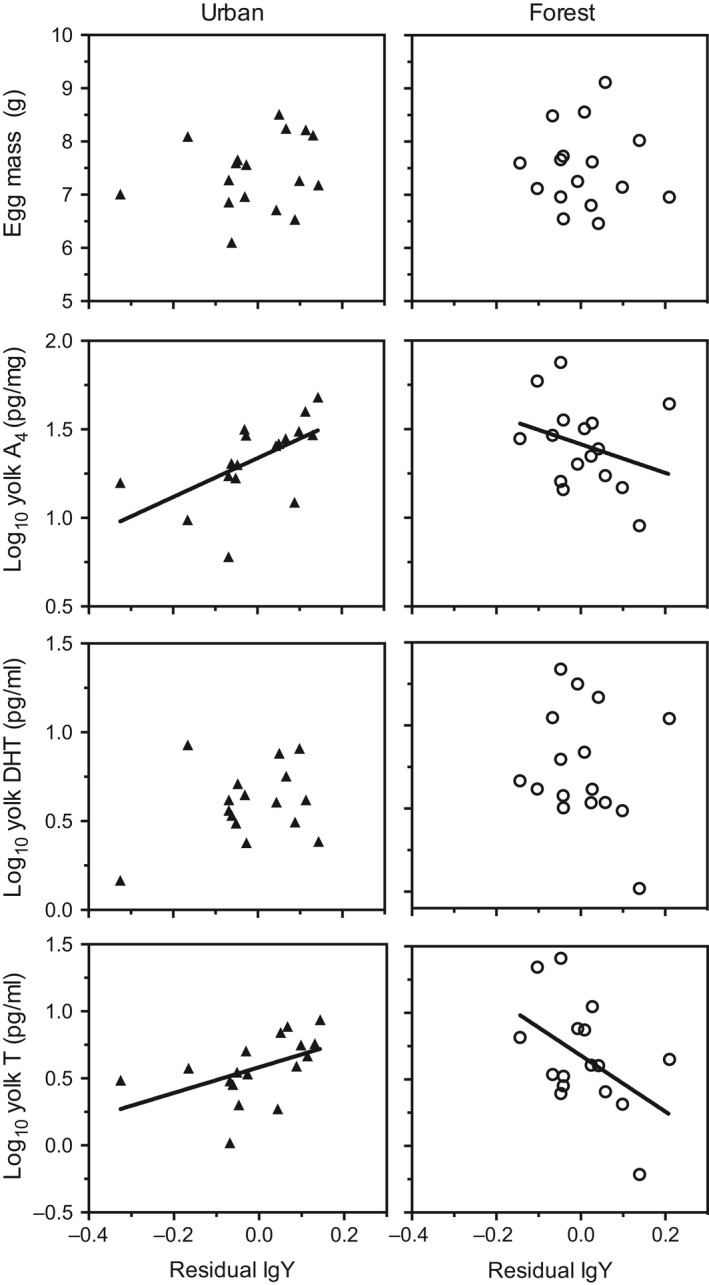
Residual immunoglobulin Y concentration (IgY) in relation to egg mass and yolk androgen concentrations in a city and a forest population of the European blackbird. Each data point represents a clutch mean. We used residual immunoglobulin Y concentrations to account for seasonal differences

The relationships of yolk A_4_ and yolk T with residual IgY concentrations (corrected for seasonal change) differed between the city and the forest populations (yolk A_4_: population × IgY interaction: *F*_1,29_ = 6.28, *p* < .05, yolk T: population × IgY interaction: *F*_1,29_ = 8.55, *p* < .01; Figure 4). The relationship was positive in the city population (yolk A_4_: *F*_1,15_ = 7.46, *p* < .05; yolk T: *F*_1,15_ = 4.56, *p* < .05, Figure 4) and absent in the forest population (yolk A_4_: *F*_1,14_ = 1.43, *p* = .25; yolk T: *F*_1,14_ = 4.11, *p* = .06; Figure 4). Association with residual IgY was absent for 5α‐DHT (*F*_1,29_ = 0.13, *p* = .72; population × IgY interaction: *F*_1,29_ = 1.36, *p* = .25; Figure 4).

## 4 DISCUSSION

Our comparison of yolk androgens and yolk immunoglobulins between city and forest European blackbirds exposed the following patterns: (a) Yolk androgen concentrations were higher in eggs of the forest than the city population; (b) among‐clutch variation of yolk androgens was also higher in the forest than city population, while within‐clutch variation was similar; (c) IgY concentrations were similar and exhibited seasonal decline in both populations; and (d) yolk A_4_ and T concentrations were positively correlated with IgY concentrations in the city, while negative tendencies were found in the forest population. These results are consistent with the hypothesis that androgen‐mediated maternal effects contribute to phenotypic differences between city and forest birds. This conclusion remains preliminary, because our study only compares one population in each habitat. Whether the differences in egg androgen levels arise from female plastic responses to different environments or reflect past direct or indirect selection on yolk androgen deposition requires additional studies.

The different yolk androgen titers may be related to fundamental ecological differences between the populations, and potentially between urban and rural habitats in general, such as population density, food availability, nest predation rates, and parasite prevalence, variables that have been shown to influence yolk androgen concentrations (Duckworth et al., 2015; Schwabl et al., 1997; Tschirren et al., 2004). Since we did not monitor these parameters in our study, we are unable to evaluate their relative contribution to the differences. However, we nevertheless briefly address breeding density here and parasite prevalence below in the context of trade‐off of immunoglobulins with androgens. Breeding density of European blackbirds is generally much higher in urban than forest populations (reviewed in Morozov, 2009). In turn, studies of diverse avian species reported positive correlation between density and yolk androgen (mainly T) levels (reviewed and meta‐analyzed in Bentz, Becker, & Navara, 2016). Therefore, we expected higher yolk androgen levels in eggs of the city than the forest population, but surprisingly we found the opposite. Apparently, factors other than density and frequency of social interactions lead either to the elevated yolk androgens in the forest population or to the lower and less variable levels in the city. The lower variation of androgen (except A_4_) levels among clutches in the city than the forest population could reflect a spatially less variable environment and/or lower genetic variation. While spatial uniformity definitely applies to our city cemetery site, reduced genetic variation is also likely since across Europe urban populations of European blackbirds show reduced genetic diversity compared to paired forest populations (Evans, Gaston, Frantz, et al., 2009).

Several lines of evidence suggest heritability of and natural selection on yolk hormone concentrations. Field and laboratory studies with wild birds showed that yolk androgen concentrations are heritable and under natural selection (Ruuskanen et al., 2016; Tschirren, Sendecka, Groothuis, Gustafsson, & Doligez, 2009); directional selection for high or low yolk androgen concentrations in domesticated Japanese quail (*Coturnix japonica*) yielded rapid divergence of titers within a few generations (Okuliarova, Groothuis, Škrobánek, & Zeman, 2011) and indicates matrilineal inheritance (Tschirren et al., 2016)*;* and artificial selection for behavioral traits such as bold and shy personalities in Great tits (*Parus major*) resulted in increasing, respectively, decreasing yolk androgen concentrations over the laying sequence (Groothuis, Carere, Lipar, Drent, & Schwabl, 2008). These results prompt us to propose that differential environments operating in urban versus rural populations lead to coupled changes in yolk androgen concentrations and behavior, the mechanistic link being the wide‐spread and well‐established organizational actions of steroid hormones on brain function during development of vertebrates (Adkins‐Regan, 2012; Fowden & Forhead, 2009; McCarthy, 2010; O'Connor & Barrett, 2014). Low rates of natal dispersal and high breeding site fidelity in urban compared to rural blackbirds (Greenwood & Harvey, 1976; Jankowiak, Wysocki, & Greño, 2016; Samaš et al., 2013) likely favor the selection for hormone‐mediated maternal effects on the phenotype of the adult offspring because of the consequent correlation of maternal and offspring environment.

Population and sex interacted to influence yolk T, with lowest yolk T levels detected in female eggs in the city. Sex‐specific, differential exposure to yolk androgens has been reported for some species (Badyaev, Acevedo Seaman, Navara, Hill, & Mendonca, 2006; Duckworth et al., 2015; Petrie, Schwabl, Brande‐Lavridson, & Burke, 2001), as have been sex‐specific effects (Ruuskanen & Laaksonen, 2010; Sockman, Weiss, Webster, Talbott, & Schwabl, 2007; Tschirren, 2015; von Engelhardt, Carere, Dijkstra, Groothuis, 2006). The large difference between the populations in exposure levels of female blackbirds to T predicts female phenotype to be more strongly impacted by maternal T than male phenotype. Common garden experiments with individuals of our blackbird populations demonstrated differences in timing of reproduction, expression of traits associated with migration, adrenocortical stress response, and personality (Costantini et al., 2014; Miranda et al., 2013; Partecke & Gwinner, 2007; Partecke et al., 2006, 2004), with some of these differences, for example tendency to express a migratory phenotype, being sex‐specific (Partecke & Gwinner, 2007). In this context, it is noteworthy to mention that experimentally enhanced yolk T exposure increased natal dispersal distance in great tits (Tschirren, Fitze, & Richner, 2007) and one could speculate that low exposure to androgens in the city blackbird population in combination with sex‐linked responsiveness (Tschirren, 2015) results in sex‐biased reduced migratory propensity. Experimental manipulations need to determine if one of the differing androgens plays a dominant role (Hegyi et al., 2011) in such effects or if ratios of the different androgens are critical in the development of population and sex differences.

Due to expected higher prevalence of parasites in forest than urban blackbirds (Evans, Gaston, Sharp, et al., 2009; Geue & Partecke, 2008), we predicted the eggs of forest females to show higher IgY concentrations than those of city females and to show an increase with progress of the breeding season. We found, however, no difference in IgY levels between the populations and a seasonal decrease in both populations. This absence of a population difference in IgY titers could be the result of yearly variation in parasite prevalence across sites; the similar decline with season in both populations may indicate seasonally changing conditions across environments resulting in a similar constitutive transmission of IgY into eggs.

The analyses of yolk androgens and immunoglobulins in the same eggs allow us to assess trade‐off and integration of maternal effect mediators. Different maternal egg components, that is, hormones and antibodies or antioxidants, should ideally be integrated to benefit the female and to generate an optimally prepared offspring phenotype for a given environment. Such balance might, however, be incomplete and/or constrained by antagonistic processes operating in the mother (Postma et al., 2014) or the offspring (Müller et al., 2005; Sandell, Tobler, & Hasselquist, 2009) but see (Navara, Hill, & Mendonca, 2006). We found, however, population‐specific relationships between residual yolk IgY (corrected for seasonal change) and androgen (A_4_ and T, but not 5α‐DHT) concentrations, with positive association in the city and negative tendencies in the forest population (where yolk androgen levels were overall higher). Apparently, IgY and yolk androgen levels are not necessarily antagonistically coupled, resulting in trade‐offs against each other, but instead can show context‐ or population‐specific associations. Previous studies yielded mixed results regarding the relationship of yolk androgen and IgY levels (Gasparini et al., 2007; Groothuis et al., 2006; Hargitai, Arnold, Herényi, Prechl, & Török, 2009; Postma et al., 2014), and a more complex, context‐specific relationship, such as the one found here, may suggest that both antagonistic variation and co‐allocation are possible in the same species (Okuliarova et al., 2014).

## 5 CONCLUSION

Our results demonstrate clear differences of yolk androgen concentrations between European blackbirds of a city and a forest population. These might result from different environments to which females respond plastically with adjustments of yolk androgens or be a consequence of direct or indirect past selection on yolk androgen transmission. Regardless of female plasticity or past selection, the different yolk androgen levels are likely to developmentally organize and program phenotype. Future studies using a gradient of cues associated with urbanization such as anthropogenic noise or human density/interaction within the urban environment instead of using urban and forest habitats will add confidence to our present results. Moreover, the results set the stage for hormone manipulation experiments to test the hypothesis that hormone‐mediated maternal effects and genes contribute to the generation of differences in behavior, physiology, and life history traits observed between urban and forest bird populations (Watson, Videvall, Andersson, & Isaksson, 2017).

## CONFLICT OF INTEREST

None declared.

## AUTHOR CONTRIBUTIONS

JP and HS conceived the ideas, designed study, and collected data. GH, PSF, JG, JP, and HS analyzed the data. All authors contributed critically to the drafts and gave final approval for publication.

## Data Availability

The data have been deposited in the publicly accessible Dryad repository (https://doi.org/10.5061/dryad.cc2fqz632).
